# Indications and complications of inpatient parenteral nutrition prescribed to children in a large tertiary referral hospital

**DOI:** 10.1186/s13052-018-0505-x

**Published:** 2018-06-08

**Authors:** C. Mantegazza, N. Landy, G. V. Zuccotti, J. Köglmeier

**Affiliations:** 1 Great Ormond Street Hospital for Children NHS Foundation Trust, London, UK; 2 Department of Pediatrics, University of Milan, Ospedale dei Bambini Vittore Buzzi, Milan, Italy

**Keywords:** Pediatric parenteral nutrition, ESPGHAN guidelines, indications, appropriatness, complications, NCEPOD

## Abstract

**Background:**

Parenteral Nutrition (PN) is prescribed to children with intestinal failure. Although life saving, complications are common. Recommendations for indications and constituents of PN are made in the 2005 guidelines by the European Society of Paediatric Gastroenterology, Hepatology and Nutrition (ESPGHAN). The aim of this study was to establish if the indications for prescribing PN in a tertiary children’s hospital were appropriate, and to identify complications encountered. Data were compared to those published by the National Confidential Enquiry into patient outcome and death (NCEPOD) carried out in the United Kingdom in 2010.

**Methods:**

Children and newborns receiving inpatient PN over a 6 months period were entered into the study and data was collected prospectively. The appropriate indications for the use of PN were based on the ESPGHAN guidelines. Recorded complications were divided into metabolic, central venous catheter (CVC) related, hepatobiliary and nutritional.

**Results:**

A total of 303 children (67 newborns) were entered into the study. The main indications for the start of PN were critical illness (66/303), surgery (63/303) and bone marrow transplantation (28/303). The ESPGHAN recommendations were followed in 91.7% (278/303) of cases (95.5% of newborns, 90.7% of children). PN was considered inappropriate in 12/303 patients and equivocal in 13. The mean PN duration was 18 days (1–160) and the incidence of complications correlated to the length of PN prescribed. Metabolic, hepatobiliary and CVC related complications affected 74.6, 24.4, 16.4% of newborns and 76.7, 37.7 and 24.6% of children respectively.

In relation to the appropriate indications for the start of PN our results mirrored those reported by the NCEPOD audit (92.4% of newborns and 88.6% children). However, the incidence of metabolic disturbances was higher in our cohort (74.6% vs 30.4% in children, 76.7% vs 14.3% in newborns) but CVC related complications lower amongst our newborns (16,4% vs 25%).

**Conclusions:**

Although the indications for inpatient PN in children is mostly justified, there is still a proportion who is receiving PN unnecessarily. PN related complications remain common. There is a need for better education amongst health professionals prescribing PN and access to nutritional support teams to reduce unwanted side effects.

## Background

Parenteral Nutrition (PN) is used when nutrition cannot be tolerated via the oral or enteral route [1]. Since its first use in the late 1960s PN has become a well established therapy and a lifesaver in well selected hospitalised newborns and children with intestinal failure (IF) or those who fail to meet their nutritional requirements with enteral intake alone [2].

However, PN is bay far no panacea, quite invasive and associated with potentially fatal complications [3]. Central venous catheter (CVC) related infection, occlusion, central venous thrombosis, pulmonary embolism and accidental catheter removal or damage, electrolyte disturbances and renal abnormalities, metabolic bone disease, micronutrient deficiencies and liver disease are well known complications of PN [3, 4].

It is hence crucial to weigh the potential benefits against the risks to the patient, led alone the cost implications to the health care system [5].

In 2005 the European Society of Paediatric Gastroenterology, Hepatology and Nutrition (ESPGHAN) and the European Society for Clinical Nutrition and Metabolism, supported by the European Society of Paediatric Research, have published guidelines on the use of PN in children, aiming at identifying the most common and reasonable indications for the use of PN in newborns, infants and children in order to reduce its inappropriate use [6]. The authors make recommendations when to start PN and how to minimise the related complications [6].

Much is published on the use and incidence of complications occuring in adults receiving intravenous nutrition in hospital [7, 8]; however little is known in children [6].

In 2010 the National confidential enquiry into patient outcome and death (NCEPOD) conducted a retrospective survey on a random sample of patients receiving PN in hospital in the United Kingdom including children and newborns [9]. The purpose of this enquiry was to reveal areas of deficiency in the use of PN in hospitalised patients and to make recommendations how to resolve them [9]. The neonatal cohort included 264 subjects but the audit in the paediatric age group consisted of just 70 patients [9]. In the neonatal cohort 92,4% of the indications for PN were considered appropriate [9]. In the paediatric age group (1 month to 19 years) 88% of children were given PN for justified reasons [9]. Moreover the audit showed that in children complications associated with PN still frequently occur: metabolic disturbances affecting 30,4% of children and 14,3% of newborns, and CVC related complications encountered in 25% of newborns, were amongst the most common ones [9]. The need of a large scale audit of PN care in children was hence suggested [9].

The aim of this observational study was hence to get a better understanding why children in hospital are started on PN and if the indications, based on the 2005 ESPGHAN guidelines, are appropriate and to estimate the incidence of PN related complications. We wanted to compare our results to the NCEPOD audit and, if needed, develop strategies to avoid inappropriate PN prescribing in the future.

## Methods

All newborns, infants and children admitted to Great Ormond Street Hospital who started PN were identified from the pharmacy database and enrolled prospectively over a 6 months study period between October 2013 and March 2014. Patients established on home PN were excluded. Information was obtained from the PN prescription, medical, nursing and dietetic records.

### Indications for starting PN

Patient demographics, weight and height/length, referring department (medical, surgical, intensive care), underlying diagnosis, current pathology, feeding regime prior to and on PN, reason for starting PN and duration were recorded. Under-nutrition in children was defined according to the World Health Organistation criteria [10]. The indications for prescribing PN were divided according to specialty into medical and surgical, and primary or secondary IF. Primary IF was defined as requirement for intravenous nutrition due to an underlying anatomical, structural gut pathology; IF was considered secondary if PN was started as a consequence of other conditions leading to enteral feed intolerance. The indications for starting PN were classified as appropriate, inappropriate or indeterminate based on the ESPGHAN 2005 guidelines [6]. In newborns with a very low or extremely low birth weight indications for PN were based on separate guidelines applicabable to this specific group of patients [11] (Tables 1 and 2).

**Table 1:** Appropriate indications for PN in infants and children [6]

1	Infants aged 1 to 12 months: inability to achieve an adequate energy intake^a^ for more than 3 days
2	In children older than 1 year: inability to achieve an adequate energy intake^a^ for more than 5 days
3	Children not expected to meet an appropriate energy and nutrient intake for more than 7 days
4	Clinical condition leading to an absolute or relative contraindication to enteral nutrition^b^
5	In children older than 1 year: duration of PN of at least 5 days except in undernourished children

^a^adeguate energy intake defined as 60–80% of the Kcal for age [5]

^b^absolute contraindications to enteral nutrition: paralytic or mechanical ileus, anatomical disruption of the gastrointestinal (GI) tract, intestinal obstruction, necrotising enterocolitis, GI ischemia, diffuse peritonitis, bowel perforation or a state of severe shock [5, 16, 90]

Relative contraindications to enteral nutrition: gastro-intestinal bleeding, intractable diarrhea, enteric fistula, toxic megacolon, gastro-intestinal dysmotility, severe vomiting [5, 16, 90]

**Table 2:** Appropriate indications for PN in newborns [5, 11]

1	< 32 weeks of gestation and/or < 1500 g
2	> 32 weeks of gestation with IF
3	Bridging PN

The use of PN in relation to appropriateness was considered indeterminate if a judgement could not be made due to lack of enough clinical information to be able to reach a conclusion if PN was used appropriately, if EN was achievable but not used because of concerns over potential complications associated with feeding or a delay in passing a suitable enteral feeding tube, eg if the child did not cooperate with the insertion of a nasal feeding device or if jejunal feeding was required but jejunal access was unsuccesful.

### PN Complications

Bloods for vitamins (A,D,E), folate, trace elements (zinc, selenium, copper, iron and mangnesium) and ferritin were sent when patients were started on PN.

Electrolites, urea, peripheral blood glucose, carbon dioxide (CO2) and pH were monitored daily for at least 10 days; subsequent testing was based on the patients clinical needs. Liver function tests (LFTs) including alanine aminotransferase (ALT), aspartate aminotransferase (AST), alkaline phosphatase (ALP), gamma-glutamyl transpeptidase (GGT) and bilirubin, urinary sodium (uNa) and triglycerids (TG) were performed at least weekly. In children who remained on PN for more than 28 days vitamins and trace elements were checked after one month of nutritional support and continued to be monitored every four weeks for the duration of PN.

All children were followed on a daily basis and any complications recorded.

Complications were divided into metabolic, CVC related, nutritional and hepatobiliary and defined as follows:

1. metabolic: deficiency or excess of electrolytes, hypo and hyperglycemia, hypercapnia, acid–base disturbances, rise in urea, hypertriglyceridemia and low uNa;
  electrolytes abnormalities were defined as at least two consecutive results above or below the reference value for age and divergent from baseline levels, requiring a change of PN prescription or oral/iv supplementation; calcium and magnesium were corrected for the albumin level, serum sodium was corrected in the presence of hyperglycemia [12]. Patients were monitored for hypophospathemia throughout the duration of PN, which was considered a marker of refeeding syndrome (RS) in the first 10 days of PN in malnourished children or those who had lost a significant amount of weight prior to the start of PN [13].
  Hypoglycaemia was defined as a blood glucose level of less than 3.6 mmol/l (40 mg/dl) both in newborns and in children [14, 15]. Children were considered hyperglycaemic if their blood sugare rose above 7 mmol/L (126 mg/dl) [14]. In newborns plasma glucose concentrations above 8 mmol/l (150 mg/dL) were considered pathological in accordance with the guidelines of the American Society for Parenteral and Enteral Nutrition [14].
  Blood triglyceride concentrations were checked on a weekly basis 4 h after stopping the lipid infusion; levels > 200 mg/dl in newborns, > 250 mg/dl in infants and > 400 mg/dl in children were considered pathological [16].
  uNa was considered low if < 20 Meq/L.
  A rise in urea due to renal failure or gastro-intestinal bleeding was not recorded [17, 18].
  Acid-base disturbances as a consequence of respiratory failure, sepsis and metabolic disease were not considered to be related to PN.
  Hypercapnia secondary to respiratory failure was not recorded.
2. CVC related complication: Exit site and central line associated bloodstream infections (CLABSIs), line colonisation, occlusion, thrombosis, damage, misplacement, phlebitis, extravasation and venous thrombosis; CLABSIs were defined according to the 2012 guidelines of Centers for Disease Control and Prevention [19]. All organisms causing CLABSIs were recorded.
3. Nutritional complications: deficiency or excess of vitamins and trace elements after 30 days of PN and divergent from baseline values were recorded; Vitamin concentrations were measured by high pressure liquid cromatography (HPLC). Ferritin was used as a marker of iron storage in the presence of a normal C-reactive protein [20]. As ferritin syntesis is enhanced by cytokines, high values were not considered due to iron overload when inflammation was present or the inflammatory markers were elevated [20].

In particular, if data were available, we followed ESPGHAN guidelines for reference values:

- we defined a high vitamin E level as greater than 3.5 mg/dl and low Vitamin E levels < 1 mg/dL and < 0.5 mg/dl in premature infants and children respectively [6, 21, 22].
- 300–800 microG/L were considered normal reference values for vitamin A in children older than 6 months of age. Serum concentrations below 200 microg/L were considered to indicate deficiency in premature infants [6].

We used reference values obtained from the ESPGHAN guidelines considered normal for sex and age if no data was available from our laboratory.

4) Hepatobiliary complications: any prolonged (at least two weeks) rise in LFTs not explained by other causes (eg medication, metabolic disorder, haemolysis, surgical obstruction) 1.5 × above the normal value for age was recorded. Hyperbilirubinaemia was defined as > 50 micromol/L.

The study was approved by the Ethical Committee of Great Ormond Street Hospital and informed consent was obtained from parents or legal guardians of the patients. All data collected were analysed using Microsoft Access and Excel.

## Results

### Population characteristics

Over a 6 months period PN was prescribed to 318 newborns and children; fifteen patients were excluded as already established on home PN. A total of 303 were hence included in the study; 236 (77.9%) were older than 1 month of age, 67 (22,1%) were newborns; the median age was 38 months (range 0-223), 54.5% were females, 89 patients were malnourished (29,4%).

Only 17 patients did not have a pre-existing diagnosis, 90 children suffered from congenital heart disease, 68 had a diagnosis of malignancy (54,4% leukemia, 46,6% solid tumour) and 41 had a known pathology of the GI tract. All the underlying diagnoses are listed in Table 3.

**Table 3:** Underlying diagnosis

Pre-existing diagnosis	No	Percentage
Cardiac disease	90	29,7
Malignancy	68	22,4
GI pathology	41	13,5
Immune dysfunction	20	6,6
No pre existing pathology	17	5,6
Metabolic disorder	16	5,3
Prematurity	16	5,3
Neurological abnormality	15	5,0
Syndrome	7	2,3
Endocrine disorder	3	1,0
Congenital bone diseases	3	1,0
Lung disease	3	1,0
Rheumatological disorder	3	1,0

Children were referred for PN from medical (34%, majority oncology 57/103), surgical (35%, majority cardiothoracic 81/106) and Intensive Care Units (31%). Main indications for prescribing PN were critical illness (66/303), pre, peri and post surgery nutritional support (63/303) and bone marrow transplantation (28/303).

The majority of children (57.4%) who were prescribed PN had secondary IF as a consequence of an acute or chronic medical condition. Multi-organ, respiratory, renal or heart failure, shock, sepsis and macrophage activation syndrome were the most common causes (21.8%) followed by bone marrow transplantation (9.8%) or feed intolerance due to chemotherapy (8.2%) or radiation induced mucositis. The indications for PN are listed in Table 4.

**Table 4:** Indications for PN

Indication	No	Percentage
Critical illness	66	21.8
Pre-peri-post surgery nutritional support	63	20.8
Bone marrow transplantation	28	9.2
Feed intolerance	25	8.2
NEC	22	7.3
Intestinal resection in congenital malformation of the GI tract	21	6.9
Mucositis due to chemotheraphy	19	6.3
Other intestinal resections	10	3.3
Condition associated with malabsorption	9	3.0
Decompensation in patients with metabolic condition	7	2.3
Organ transplant	6	2.0
Post surgical ileus	4	1.3
Autoimmune disorder associated with intestinal failure	4	1.3
GI dysmotility	4	1.3
Miscellaneus	4	1.3
Post operative nutritional support	3	1.0
Malnourishment	3	1.0
Meconium aspiration	2	0.7
Acute pancreatitis	2	0.7
Heart cachexia	1	0.3

At the end of the study all but 15 patients were weaned off PN. The mean duration of PN was 18 days (range from 1-160 days), 55 patients (18,2%) had more than 28 days of intravenous support.

### Appropriatness of indications for prescribing PN

PN was considered appropriate in 278/303 (91,7%), inappropriate in 12 (4,0%) and indeterminate in 13 (4,3%) patients.

PN was defined inappropriate in 5 non malnourished children older than 12 months who had less than 5 days of PN where it was not anticipated at the start that PN would be required for longer; moreover four infants were given PN despite a functional GI tract and one was kept nil by mouth and given PN for one day for query NEC but started again on feeds the following day. Finally two term newborns were precribed PN unnecessarily as they were able to tolerate >80% of their requirements enterally when PN was started and PN was only given for one day.

According to age PN was defined as appropriate in 95,5% of newborns and in 90,7% of infants and children.

Our results were similar to the data reported in the NCEPOD audit [9] Table 5.

**Table 5:** Appropriatness of PN divided by age and compared to the NCEPOD audit

	Current Audit		NCEPOD	
	Newborns	Infants/children	Newborns	Infants/children
	No, Percentage	No, Percentage	No, Percentage	No, Percentage
Appropriate	64, 95.5	214, 90.7	244, 92.4	62, 88.6
Indeterminate	1, 1.5	12, 5,1	4, 1.5	/
Inappropriate	2, 3.0	10, 4.2	16, 6.1	8, 11.4

### Complications

A total of 239/303 (78,9%) patients developed complications as a consequence of PN; the mean duration of PN in these children was 22 days (range 2-160) whereas those who experienced no complications received PN for a mean of 5 days (range 1-23). All patients who had PN for more than 28 days developed complications.

Metabolic complications were the commonest occuring in 76.2% of patients: hyperphosphatemia was the most frequent (111 patient, 36.6%), followed by hypermagnesemia (31,4%) and hypercalcemia (29,7%). In particular hyperphosphatemia developed in 8/89 malnourished patients. Hypophosphatemia was seen in 84 patients (27,7%) and 66/84 (59,9%) developed it in the first 10 days of PN administration. RS was blamed in 13/66 patients as they were malnourished or had lost a significant amount of weight prior to the start of PN. However, most malnourished patients (85.4 %) did not develop hypophosphatemia in the first days after being started on intravenous support.

Hyperglycemia and hypoglycemia affected 32 (10.6%), and 9 (3.0%) of all children respectively. Twenty-seven (8.9%) children developed hypercapnia. Acid-base disturbances were noted in 55 children, 36/303 (11.9%) developed acidosis.

All metabolic complications are listed in Table 6.

**Table 6:** Metabolic complications

Metabolic complication	No	Percentage among all patients
Hyperphophatemia	111	36,6
Hypermagnesemia	95	31,4
Hypercalcemia	90	29,7
Hypophosphatemia	84	27,7
Rise in urea	74	24,4
Hypokalemia	70	23,1
Hypophosphatemia in the first 10 days	66	21,8
Hypernatremia	36	11,9
Acidosis	36	11,9
Hyponatremia	35	11,6
Hyperkalemia	32	10,6
Hyperglycemia	32	10,6
Hypercarboxinemia	27	8,9
Hypomagnesemia	25	8,3
Hypertrigliceridemia	21	6,9
Hypocalcemia	19	6,3
Alcalosis	19	6,3
Hypercloremia	14	4,6
Hypocloremia	13	4,3
Low urinary sodium	12	4,0
Hypoglycemia	9	3,0

Hepatobiliary complications were found in 106 patients (35,0%). A rise in ALT was the commonest affecting 82/303 (27.1%). Elevation of GGT occured in 38 children (12,5%); an abnormal bilirubin in 31 (10,5%). Rise in ALP was documented in 14 patients. Just 5 had an elevated AST. Twenty-one patients with a derangement of LFTs had a concomitant line infection, 45/106 (42,5%, 14.8% of the total population) had a rise in more than one liver parameter. The mean duration of PN in patients who developed hepatobiliary complications was 31 days versus 12 days in children who did not experience any alteration in LFTs.

CVC related complications affected 69 (22,8%) children, the commonest being blockage of the line (36, 11.6%). Thirty-one children (10.2%) developed a line infection (no child had more then one episode). The principle organism involved was coagulase negative staphylococcus grown in 17/31 (54,8%) blood cultures followed by Streptoccocus species (5/16,1%) and Candida species (3, 9.7%). Line infections with gram negative organisms occured in 4 children (12.9%) and was caused by Enterobacteriacae (2/4), *Escherichia coli* (1/4) and Pseudomonas Aeruginosa (1/4). The mean duration of PN in children who developed a line infection was 37 days versus 17 days in children who did not develop CLABSIs. The central venous catheter was subsequently removed and replaced with a new line in 11/31(15.9%) patients. Colonisation of the line was found in 9/31 patients, mostly with Coagulase negative Staphylococcus (5/9, 55.6%). Four children experienced a catheter breakage which required repair.

Thirty-five/303 (11,6%) children developed nutritional complications. Of the 55 children who received PN >28 days, 37 had nutritional bloods performed. 35/37 (94,6%) showed a deficiency or excess in the levels of fat soluble vitamins and/or trace elements. The most common abnormal results seen were a rise in vitamin A and E levels and low levels of selenium with 27.3%, 25.5% and 29.1% of patients affected respectively. All the nutritional complications encountered are listed in Table 7.

**Table 7:** Nutritional complications

Nutritional complication	No	Percentage among all patients with PN > 28 days
Vitamins		
High vitamin E	15	27,3
High vitamin A	14	25,5
High vitamin D	1	1,8
Low vitamin D	1	1,8
High folate	7	12,7
High vitamin B12	1	1,8
Trace elements		
Low Zinc	7	12,7
High Zinc	1	1,8
Low selenium	16	29,1
High selenium	2	3,6
Low copper	9	16,4
High copper	5	9,1
Low iron	4	7,3
High iron	1	1,8
High Manganese	1	1,8

According to age metabolic complications affected 74.6% of all newborns, the commonest being hyperphophatemia (33/67) and hypermagnesemia (30/67), and 76.7% of all children. Hyperglycaemia developed in 10.45% of newborns, and hypoglycemia in 6.0%. Seventeen (25.4%) of newborns and 89 (37.7%) of children showed a derangement in their LFTs.

Eleven newborns (16.4%) and 58 children (24.6%) developed CVC related complications, in particular 4 newborns had a culture positive CLABSIs.

Nutritional complications developed in 3 newborns and 32 children.

Comparison between our audit and the NCEPOD is reported in Table 8 [9].

**Table 8:** Comparison between our audit and NCEPOD

	Current Audit		NCEPOD	
	Newborns	Infants/children	Newborns	Infants/children
	No, Percentage	No, Percentage	No, Percentage	No, Percentage
Metabolic complications	50, 74.6	181, 76.7	63, 30.4%	10, 14.3
CVC related complications	11, 16.4	58, 24.6	56, 25.0	/

## Discussion

Feeding children with a non functioning gut intravenously or those who are unable to meet their nutritional requirements enterally is a well established practice. However, PN is by far no panacea and its use may cause serious complications to the patient and put a huge financial burden to the health care system [3, 4, 23], The 2005 ESPGHAN guidelines make recommendations when PN should be prescribed to children [6]. In 2010 the NCEPOD conducted a national retrospective audit in the UK on adults and children receiving PN: indications for PN were considered appropriate in 92.4% of newborns and in 88.6% of children aged >1month [9]. Complications were demonstrated to be common. Metabolic disturbances occured in 30,4% of children and 14,3% of newborns of which 25% also developed CVC related complications [9].

The NCEPOD audit revealed suboptimal care in the administration of PN in newborns and children and pointed out the importance of the Nutrition Support Team (NST) [9]. However, as only 70 children were included in the data analysis, a larger scale audit of PN care in children was recommended [9].

The findings of our study mirror those of the NCEPOD; yet our data was collected prospectively and based on a much bigger cohort of 236 children. Although our neonatal group was smaller (67 versus 264 NCEPOD) we had similar results in this age group as well. In our cohort the use of PN was appropriate in 95.5% of newborns and 90.5% of children (aged 1 month-18 years). We observed a high complication rate with 78.9% of patients being affected. Similar to the NCEPOD audit, metabolic disturbances were the most frequently occuring problem and exceeded the percentage of children affected in the 2010 retrospective NCEPOD study. However, CVC related complications were less common in our population compared to the NCEPOD audit. Amongst our patients 16.4% experienced problems with their catheter compared to 25.0% in the NCEPOD group. However, complications were poorly defined in the NCEPOD study and comparison between the two studies is hence difficult. Of note is the fact that PN continues to be prescribed inappropiately in a percentage of children which may lead to serious or even life-threatening complications. All effort should hence be made to avoid the use of PN if clinically not clearly indicated.

### Inappropriate prescribing of PN

In our cohort PN was inappropriately prescribed to children with a functional gut when enteral feeds were not tried vigorously enough and in situations associated with a temporary feed intolerance. In particular in children older than 12 months of age, with a good nutritional status, post operative PN is considered beneficial only if the child is expected to remain nil by mouth for more than 7 days and when PN is administered for at least 5 days, otherwise the risk associated with the administration outweighs the benefits [9, 24–26].

### Main Metabolic complications: hyperphosphatemia, hypercalcemia, hypermagnesemia, hypophosphatemia

The commonest metabolic complications seen in our patients were an elevated phosphate, magnesium and calcium. These minerals have a crucial role in the cellular physiology of the neuromuscolar and cardiovascular systems [27]. Derangement can lead to significant pathologies including respiratory failure, disruption of the cardiac conduction system with arrhythmias, complete heart block, and in severe cases cardiac arrest and coma [27–35]. In the oncology children rumour lysis syndrome may have contributed to these abnormal results [29]. An overawareness of RS could have also led to overprescribing of electrolytes. Given that only a small number of malnourished patients (9.0%) developed hyperphosphatemia this appears unlikely though. Hypophosphatemia was also commmonly observed in our population with 23.1% of children/newborns affected. Hypophosphatemia is associated with respiratory insufficiency and decreased myocardial contractility, rhabdomyolysis, hemolysis, impaired platelet and white blood cell function, and neurological abnormalities including seizures [36, 37]. Hypophosphatemia during the first 7-10 days on PN may also be a consequence of RS, a potentially lethal condition which occurs in starved, malnourished or ill metabolically stressed patients when too much nutrition is provided initially [13, 38]. Based on this definition we found that up to 21.8% of our patients were affected but amongst them just 19.7% were malnourished (4,3% of the total population). A study from 2016 on RS in adults receiving artificial support reported only a 2% incidence. However RS was more rigorously defined not only by the finding of hypophasphatemia [39]. Data collected retrospectively on children in the same year showed that hypophosphatemia, hypokalemia and hypomagnesimia were frequent in children receiving inpatient PN before and during the first four days after starting PN [40] . Malnourished children in this cohort were found to be more prone to develop low levels of potassium, wheras patients who received a high intravenous protein load had a higher incidence of hypomagnesaemia [40].

More than 15 % of newborns who were entered into the NCEPOD audit experienced low phosphate levels [9]. However, the authors do not comment whether this was felt to be a consequence of RS or not. In our cohort a large proportion of malnourished patients (85.4%) did not develop hypophosphatemia in the first days after starting PN. It is standard practice in our hospital to give extra phosphate at the start of PN in children who are at high risk of developing RS. The calorie intake is built up gradually over a few days whilst children are carefully monitored which may explain the lower incidicence of hypophosphataemia in our patients.

### Hyperglycemia

An excess in serum glucose levels affected 10.6% of our patients. Hyperglycaemia is associated with a higher infection rate [41], can lead to overfeeding [42, 43] and even death [44–46]. A glucose intake exceeding the maximal oxidation rate promotes fat synthesis and deposition [47, 48] with a large increase in CO2 production [49]. Hypercapnia, which was found in 8.9% of our patients, is compensated by increasing the respiratory rate or depth of breathing, which is well tolerated by stable patients but may lead to complications in patients with respiratory compromise [49]. In addition the overfeeding of glucose can have a negative impact on liver function, lead to hepatic steatosis and a rise in serum transaminases [50, 51]. Neonates receiving PN are particularly vulnerable to the consequences of hyperglycemia which is positively correlated to high morbidity and mortality, especially in premature and low birth weight infants [52, 53]. As this age group is prone to developing low blood sugar levels large amounts of glucose are often administered to neonates receiving PN which can easily cause hyperglycemia if children are not monitored closely [52, 53]. Unlike the NCEPOD audit, which showed a >20% prevalence of hyperglycemia in newborns, we only saw elevated glucose levels in 10.45% of our newborns. Although we were encouraged by these results there is still a significant amount of work required to improve blood sugar control in these most vulnerable children. The guidelines of the American Society for Parenteral and Enteral Nutrition suggest that newborns should receive intravenous lipids when dextrose is infused, as glycerol is the predominant gluconeogenic substrate [54, 55] and to consider insulin in cases of prolonged hyperglycemia [56].

### Hepatobiliary complications

In pediatric patients receiving PN, various definitions of PN-associated cholestasis (PNAC) and intestinal failure–associated liver disease (IFALD) are used in the literature [57]. There is hence a great variation in the prevalence of hepatic complications reported ranging from 7.4% to 84% in children [58–60]. In infants the prevalence of PNAC appears higher with 30%–70% being affected [58, 61, 62]. In keeping with the definition of the Hepatology working group of the Bristish Society of Paediatric Gastroenterology, Hepatology and Nutrition we considered IFALD as the elevation of LFTs including AST, ALT, GGT, ALP ×1.5 above the reference range for age and a bilirubin level > 50 micromoli/L for [61]. The BPSGHAN position paper consideres persistently elevated LFTs for a minimum of 6 weeks abnormal [61. However a ESPGHAN position paper from 2015 defines early IFALD as the presence of hyperbilirubinemia (>20 mmol/L) for at least 2 to 4 weeks [63]. The authors state further, that children with IF but without IFALD frequently have an isolated increase in transaminases or a moderate increase in GGT (usually <150 IU/L) [63]. Therefore, we decided to considered a time period of 14 days pathological.

We found deranged LFTs in 106 patients, 35.0% of our population. Amongst those, 45/106 (42,5%, 14.8% of the total population) experienced an elevation of more than one test. We found the occurence of abnormal LFTs was correlating to the duration of PN and the longer PN was given the higher the incidence. Our results are in keeping with other reports from the literature. As 21 patients had a concomitant line infection which is a well documented cause of liver inflammation our numbers may have, however been falsely elevated [64].

Early signs of steatosis after two weeks of PN are a mild to moderate elevation of AST and ALT, whilst serum alkaline phosphatase and bilirubin are usually not raised [58]. Cholestasis on the other hand is associated with elevated serum concentrations of ALP, GGT and bilirubin [58]. The majority of our patients (82/303; 27.1%) had an isolated rise in ALT suggestive of an inflammatory response of the liver or early steatosis. Cholestasis was less common. Steatosis is potentially reversible even if PN is continued, whilst cholestasis may progress to cirrhosis and liver failure if weaning off PN is not possible [58]. However, a significant number of our patients demonstrated a rise in GGT and bilirubin affecting 38 (12,5%) and 31 (10,5%) respectively. Steatosis can be a consequence of intravenous overnutrition [47, 48]. Unfortunately we did not have data available on the amount of non nitrogen calories provided per Kcal of body weight and we can hence not comment if too much nutrients could be held responsible.

### CVC related complications

Central venous catheters are integral to pediatric PN administration and their benefits should be carefully balanced against the risk they pose [65–68]. CVC related complication were less common in our population (16.4%) compared to the NCEPOD audit (25 %) .On the other hand, central line infections were much more common in our cohort of infants (4/67) in comparison to the relatively low numbers (5/264) in the NCEPOD audit. There is clear need in our very young patients to reduce infection rates.

CLABSIs are a known complication of PN administration [69, 70]; a prolonged catheter dwell time is associated with an increased risk for infection and the microorganisms usually involved are Gram positiva bacteria [71, 72]. CLABSIs account for significant morbidity, mortality and financial burden to the health care system [72].

In our study 31children (10.2%) developed a central line infection. The mean duration of PN administration was longer in these children than in the group which did not develop any CVC related infection (37 days vs 17 days). Gram positive microorganism were responsible for altmost 71% of infections, followed by gram negative bacteria (12.9% ) and candida species (9.7 %). Our results are simlar to those found in the literature [69–72].

Central venous catheter blockage affected 11.6% of children. Similar numbers are reported in patients on PN within 1 to 2 years after catheter placement (14% to 36%) [73]. Catheter occlusion can negatively impact on patient care: it can lead to a delay or cancellation of medical procedures, potentially interrupt the administration of intravenous medication and nutrition and has a risk of CLABSIs which in turn may lead to catheter removal [74, 75]. We were surprised about the relatively high incidence of early catheter blockage. However, many of the patients requiring chemotherapy for malignancies and those post bone marrow trabsplantation require multiple blood products and their catheter is accessed frequently for a large number of drugs, which may account for the number of catheter related problems encountered.

### Nutritional complications: high vitamin A and E and low selenium levels

In children who received PN longer than 28 days, nutritional complications occured in 35/55 patients. As 18/55 did not have bloods for micronutrients sent the figure may even be higher. The adequate provision of vitamins and trace elements intravenously can be challenging and we were hence not surprised by the large number of patients affected. The exact optimal amount of individual vitamins and trace elements children and neonates require remains a matter of debate, and only scanty evidence is available from the literature in the last 20 years [76]. In particular, little is know about the micronutrient requirements in children with acute and chronic disease. theri needs may be significantly different to those of healthy children [6].

The ESPGHAN guidelines state that the actual amount of vitamins delivered to the patient through PN may be much lower than the intended dose as vitamins degradate over time in the PN bag [6]. We were hence suprised to see the number of children who had high levels of vitamin A and E. In our PN compounding unit fat soluble vitamins are added to the lipid emulsion at the following concentration: retinol 230 IU per Kg, maximum dose 2300 IU, which is in keeping with the ESPGHAN guidelines of 2005; alfa-tocoferol 0.7 IU per Kg, maximum 70 IU, which is well below the recommended dose (2.8-3.5 IU/kg). Both vitamins tend to leach into the giving set which may lead to further losses [77]. We therefore expected a deficiency of these vitamins, particularly vitamin E. However, we found high levels of vitamin E and A in 27.3% and 25.5% of children recespectivley who received inpatient PN for more than one month. Both hypervitaminosis and hypovitaminosis A have been reported in patients who are fully PN dependant [78–80]; Hack et al found elevated vitamin A levels in approximately one fourth of their patients [81]. Although their results are comparable to ours, their patients received much higher doses than ours (214% of the recommended dose). Hypovitaminosis E is more frequently reported in children receiving PN [82]. In contrast, we found a large prevalence of high vitamin E levels in our group of children. We were concerned our results were falsely elevated tue to the way they were analysed in our lab. However, the method used (liquid chromatography) excludes falsely elevated vitamin E levels and our results hence appear to be genuine. No child in our study or in the study by Hack et el demonstrated any symptoms associated with vitamin A or E toxicity [81]. Multicenter studies or a national database such as the paediatric electronic British Articificial Nutrition Survey (eBANS) may be useful in identifying if children receiving PN are genuinely at risk of high fat soluble vitamin levels or whether there is a subgroup of patients who is more vulnerable.

When anlysing our trace element results we found low levels of selenium in altmost 30% of children who received PN for more than 28 days. Selenium deficiency can lead to significant complications including cardiomyopathy, arthritis, skeletal myopathy, loss of pigmentation of hair and skin and macrocytosis [83]. Increased transaminases and creatinine kinase have been noted in patients on long-term parenteral nutrition without selenium supplementation [83]. In children with less than 15 kg body weight, PN bags compounded by our PN pharmacy provide 25 nanomol/kg/day and in those with more than 15 Kg 4 nanomol/kg/day. Both are in keeping with current ESPGHAN guidelines. However, the precise requirements for trace elements in parenteral provision remain still a matter of debate and alterations in the blood levels of children on PN as in our group may hence be not suprising [76]. The results should be interpreted with caution though, as plasma selenium levels reflect dietary intake rather than selenium stores or bioavailability [84]. Selenium-dependent enzyme activity in tissue and body fluids in addition to serum selenium levels may hence be a better measure [6, 85].

Our results suggest thats methods aiming to detect micronutrient stores and bioavailability should be developed to detect a truly deficient state or potentially toxic levels prior to manifestations of clinical symptoms.

## Conclusions

Despite the advancements made since PN was first prescribed to children in the late 1960s, PN is still prescribed inappropriately and complications occur frequently in the hospital setting. There is clearly still work to be done to improve inpatient prescribing of PN to children. The indications for PN have evolved over the last forty years. Initially PN was prescribed to patients with a primary pathology of the GI tract [86]. However, in recent years PN is now commonly used for nutritional support in other conditions [16]. This is strongly supported by our data, as the majority of children received PN for a other conditions than a primary GI pathology (61.7% non GI vs 38.3% GI). It is still not clear how the critically ill child should be fed and both optimal route of feeding (enteral versus parenteral) and timing for the start of nutritional support is subject to debate [87]. Given that more and more children are admitted to paediatric intensive care units urgent randomized controlled trials are needed to guide physicians in the decision making process. Moreover consultation of a NST has been shown to reduce the number of children receiving short term PN unnecessarily. When a NST is consulted enteral feeds are more frequently started early and the incidence of catheter related and metabolic complications reduced. Utilisation of an NST therefore reduces the burden of care and is cost effective [88, 89]. Our hospital is staffed with a NST, and children receiving PN are reviewed once a week. However, it is currently not routine practice to consult this team prior to the start of PN, which may lead to overprescribing of PN. Studies on adults have shown that mandatory involvement of the NST prior to the start of PN reduced the number of inappropriate PN prescriptions [89]. We will therefore propose to our hospital executive board that the same policy should be put in place in our unit in the future. Moreover, given the high percentage of children developing complications, it might also be of benefit to review these most vulnerable of patients more frequently. Future staffing numbers should take this into consideration.

## Abbreviations

ALP: Alkaline phosphataseALT Alanine aminotransferase
AST: Aspartate aminotransferase
CLABSIs: Central line associated bloodstream infections
CVC: Central venous catheter
eBANS: Paediatric electronic British Articifical Nutrition Survey
ESPGHAN: European Society of Paediatric Gastroenterology, Hepatology and Nutrition
GGT: Gamma-glutamyl transpeptidase
HPLC: High pressure liquid cromatography
IF: Intestinal failure
LFTs: Liver function tests
NCEPOD: National Confidential Enquiry into patient outcome and death
NST: Nutrition Support Team
PN: Parenteral Nutrition
RS: Refeeding syndrome
TG: Triglycerids
uNa: Urinary sodium
